# Molecular Influence of Resiniferatoxin on the Urinary Bladder Wall Based on Differential Gene Expression Profiling

**DOI:** 10.3390/cells12030462

**Published:** 2023-01-31

**Authors:** Ewa Lepiarczyk, Łukasz Paukszto, Marta Wiszpolska, Elżbieta Łopieńska-Biernat, Agnieszka Bossowska, Mariusz Krzysztof Majewski, Marta Majewska

**Affiliations:** 1 Department of Human Physiology and Pathophysiology, School of Medicine, Collegium Medicum, University of Warmia and Mazury in Olsztyn, 10-082 Olsztyn, Poland; 2 Department of Botany and Nature Protection, Faculty of Biology and Biotechnology, University of Warmia and Mazury in Olsztyn, 10-727 Olsztyn, Poland; 3 Department of Biochemistry, Faculty of Biology and Biotechnology, University of Warmia and Mazury in Olsztyn, 10-719 Olsztyn, Poland

**Keywords:** resiniferatoxin, RNA-Seq, urinary bladder wall, animal model

## Abstract

Resiniferatoxin (RTX) is a potent capsaicin analog used as a drug for experimental therapy to treat neurogenic disorders associated with enhanced nociceptive transmission, including lower urinary tract symptoms. The present study, for the first time, investigated the transcriptomic profile of control and RTX-treated porcine urinary bladder walls. We applied multistep bioinformatics and discovered 129 differentially expressed genes (DEGs): 54 upregulated and 75 downregulated. Metabolic pathways analysis revealed five significant Kyoto Encyclopedia of Genes and Genomes (KEGG) items (‘folate biosynthesis’, ‘metabolic pathways’, ‘sulfur relay system’, ‘sulfur metabolism’ and ‘serotonergic synapse’) that were altered after RTX intravesical administration. A thorough analysis of the detected DEGs indicated that RTX treatment influenced the signaling pathways regulating nerve growth, myelination, axon specification, and elongation. Many of the revealed DEGs are involved in the nerve degeneration process; however, some of them were implicated in the initiation of neuroprotective mechanisms. Interestingly, RTX intravesical installation was followed by changes in the expression of genes involved in synaptic plasticity and neuromodulation, including 5-HT, H2S, glutamate, and GABA transmission. The obtained results suggest that the toxin may exert a therapeutic, antinociceptive effect not only by acting on TRPV1 receptors.

## 1. Introduction

Resiniferatoxin (RTX) is an ultrapotent capsaicin analog naturally occurring in the latex of several members of *Euphorbia* plants. The main mode of RTX action consists of blocking the transient receptor potential vanilloid type 1 (TRPV1) nonspecific Ca^2+^ channel [1], expressed at the highest level in a subpopulation of primary afferent sensory neurons involved in nociception [2]. Because of its analgesic properties, the toxin has been used in humans for the experimental treatment of several dysfunctions associated with enhanced sensitivity, such as postoperative or postinjury ophthalmic pain [3], skeletal muscle pain [4], and premature ejaculation [5]. Moreover, RTX has been applied in clinical trials for cancer [6,7] and osteoarthritis pain therapy [8] and was even proposed as a possible agent blocking TRPV1-positive pulmonary pathways in patients with advanced COVID-19 disease [9].

As a potent blocker of primary afferent sensory neurons that is able to weaken or even stop an exaggerated C-fiber input-dependent sacral micturition reflex, RTX has received an extensive pharmaceutical interest for the effective management of lower urinary tract symptoms (LUTSs), predominantly those associated with detrusor instability or hyperreflexia, such as neurogenic detrusor overactivity, idiopathic detrusor overactivity, and urgency [10,11,12]. Numerous recent publications have proven that RTX is effective in the treatment of a range of storage LUTSs [11,12,13,14]. Additionally, a systematic review performed by Liu et al. [15] revealed that RTX produced the most effective improvement in the therapy of interstitial cystitis/bladder pain syndrome (from the eight intravesically instilled agents investigated in the experiment). Although the main mechanism of RTX action seems to be clear, the results of some studies suggest that its influence may not be limited to sensory fibers and TRPV1 [16,17,18]. The discovery of specific dysregulated genes may shed new light on the full impact of the drug on the treated organs. Therefore, we decided to explore the specific molecular pathways that underlie the RTX mechanism of action; thus, for the first time, we performed a targeted bioinformatic analysis of control (CTR) and RTX-treated urinary bladder wall samples. We conducted the experiment on domestic pigs because they are considered one of the best animal models for use in biomedical research (due to their anatomical, physiological, and genetic resemblance to humans) [19,20,21]. Moreover, the relevant size of the animal and anatomical structure of the urinary tract allowed us to mimic the intravesical route of RTX administration used in humans.

## 2. Materials and Methods

### 2.1. Laboratory Animals

The study was performed on 12 juvenile (8–12 weeks old; 15–20 kg bodyweight (b.w.)) female Large White Polish pigs. The animals were kept under standard laboratory conditions. They were fed standard fodder (Grower Plus, Wipasz, Wadąg, Poland) and had free access to water. To ensure adequate acclimatization, the pigs were transported from the breeder to the animal quarters 5 days before the scheduled procedure. The pigs were divided into two groups. Six pigs served as controls (CTR) and were treated with intravesical instillation of a 5% aqueous solution of ethyl alcohol (60 mL). Another group of 6 pigs was treated with RTX (product code: AG-CN2-0534, AdipoGen Life Sciences, Hamburg, Germany) by intravesical instillation of the toxin (500 nmol per animal in 60 mL of 5% aqueous solution of ethyl alcohol) to mimic the dose and route of its administration used in humans. Both the alcohol and RTX solutions were given under anesthesia. First, all the animals were pretreated with atropine (Atropinum Sulfuricum, Polfa, Warsaw, Poland, 0.05 mg/kg b.w., s.c.) and azaperone (Stresnil, Janssen Pharmaceutica, Beerse, Belgium, 2.5 mg/kg b.w., i.m.). After that, buprenorphine (Bupaq, Richter Pharma AG, Wels, Austria, 20 µg/kg b.w., m.c., i.m.) was given to abolish the visceral pain sensation. Thirty minutes later, anesthesia and analgesia were induced by intramuscular injection of ketamine (Bioketan, Vetoquinol, Gorzów Wielkopolski, Poland, 10 mg/kg b.w.) and xylazine (VetaXyl, Vet Agro, Lublin, Poland, 0.15 mg/kg b.w.) and by propofol (Propofol-Lipuro, B. Braun Melsungen AG, Vienna, Austria, 10 mg/kg b.w.,) given intravenously in a slow, fractionated infusion. The depth of anesthesia was monitored by testing the corneal reflex.

One week after the administration of the aqueous solution of ethyl alcohol or RTX, all the pigs were once again premedicated and deeply anesthetized (the drugs, their doses, and routes of administration were analogous to those previously used), and a midline laparotomy was performed. The urinary bladder was gently exposed in each animal and cut out for transcriptome analysis (the samples of the urinary bladder wall used in this experiment were collected from the middle part of the ventral side of the bladder). The samples were taken in vivo to ensure the acquisition of high-quality and -purity RNA for transcriptome sequencing. Next, all the pigs were euthanized with an overdose of sodium pentobarbital (Euthasol, LeVet B.V., Oudewater, Holland, 140 mg/kg).

### 2.2. RNA Extraction, Library Construction, and Sequencing

Within both the experimental and CTR groups, total RNA was isolated from the collected parts of the urinary bladder wall. RNA was isolated from the samples taken from all the investigated pigs (n = 12). Based on the manufacturer’s instructions (Thermo Fischer Scientific, Durham, NC, USA), total RNA was extracted using a mirVanaTM kit. The quantity and the quality of the total RNA isolates were evaluated using a Bioanalyzer 2100 (Agilent Technologies, Santa Clara, CA, USA). The samples with the highest RIN values and concentrations were selected for RNA-Seq library construction. Each sample was prepared with 1 μg of total RNA using an Illumina TruSeq mRNA LT Sample Prep kit (Illumina, Inc., San Diego, CA, USA). The first step involved the purification of mRNA molecules using poly-T-attached magnetic beads. Next, the mRNA was cut into small fragments with divalent cations. The cleaved RNA pieces were amplified into first-strand cDNA using SuperScript II reverse transcriptase (Invitrogen, Waltham, MA, USA) and random primers. In the upstream step, second-strand cDNA synthesis using DNA Polymerase I and RNase H was performed. The purified products of the PCR reactions were enriched, and the final cDNAs libraries were constructed. The RNA-Seq libraries were quantified using qPCR according to the qPCR Quantification Protocol Guide (KAPA Library Quantification kits for Illumina Sequencing platforms) and qualified using TapeStation D1000 ScreenTape (Agilent Technologies, Waldbronn, Germany). Indexed libraries were then sequenced using the NovaSeq6000 platform (Illumina, San Diego, CA, USA). The RNA-Seq data were submitted (https://www.ebi.ac.uk/ena, accessed on 30 September 2022) to the European Nucleotide Archive under accession no. PRJEB55778.

### 2.3. Transcriptome Sequencing, Differential Expression Profiles, and Functional Annotations

After the high-throughput sequencing process, 2 x 150 bp stranded paired-end reads were obtained. Next, the raw reads of the dataset were evaluated according to the quality control standards with FASTQC software version 0.11.7 [22]. The Illumina adaptors and low-quality reads (PHRED cut-off score < 20) were excluded during the quality control (Trimmomatic software v. 0.38) [23] based on the following criteria: all sequences were cropped to 140 bp, and 10 bp frameshifts were checked. After evaluation, the clean paired-end reads were aligned to the pig reference genome with ENSEMBL annotation (Sus_scrofa.Sscrofa11.1.104) with the STAR mapper. The following ENCODE parameters were used in the mapping procedure: -outSAMtype BAM, -SortedByCoordinate, -outFilterType BySJout, -outFilterMultimapNmax 20, -alignSJoverhangMin 8, -alignSJDBoverhangMin 1, -outFilterMismatchNmax 999, -alignIntronMin 20, -alignIntronMax 1000000, and -alignMatesGapMax 1000000. StringTie v. 1.3.3 [24] and the ballgown [25] pipeline were used to annotate and estimate the expression of porcine genes and uncover regulatory TARs. The Binary Alignment Map (BAM) files and the ENSEMBL gtf file were re-evaluated with the StringTie v. 1.3.3 pipeline to annotate the intergenic-expressed regions and uncover their regulatory properties. Ballgown software and prepDE.py script were used to prepare the counts and fragments per kilobase of transcript per million (FPKM) matrix. The DEGs were identified with the negative binomial DESeq2 method. The final results encompassed significant (adjusted *p*-value < 0.05) DEGs with known protein-coding annotation. The DEGs were transferred to the downstream functional and pathway enrichment module to be analyzed with gProfileR based on Gene Ontology (GO) [26] and the Kyoto Encyclopedia of Genes and Genomes (KEGG) [27] databases. Differentially expressed results were illustrated with the Volcano, circle heatmap, GOplot, and circlize Bioconductor R packages. The pathways illustrations were drawn with the pathview and KEGG.db R packages.

### 2.4. Real-Time PCR

The mRNA content of selected genes was determined by real-time PCR. Primers for the selected genes were designed using Primer3Plus software [28] (ELIXIR, Hinxton, Cambridgeshire, UK) based on the sequences listed in Supplementary Table S1. Total RNA was isolated using a mirVana kit following the manufacturer’s procedure (Thermo Fischer Scientific, Waltham, MA, USA), and cDNA was obtained using an Applied Biosystems™ High-Capacity cDNA Reverse Transcription Kit (Thermo Fisher Scientific, Vilnius, Lithuania, cat. no. 4374966) according to the manufacturer’s protocol. Real-time PCR was performed using Applied Biosystems™ PowerUp™ SYBR™ Green Master Mix (Thermo Fisher Scientific, Vilnius, Lithuania, Cat. No. A25780) according to the manufacturer’s protocol on a QuantStudio™ 3 Real-Time PCR System (Applied Biosystems™, Thermo Fisher Scientific Inc., Waltham, MA, USA). Briefly, each reaction contained 5 μL of master mix (2X), forward and reverse primers (1000 nM each), 10 ng of cDNA, and an appropriate volume of nuclease-free water to a final volume of 10 μL. Reactions were performed in four technical replicates for each biological sample. The expression of each gene was calculated using the comparative Pfaffl method [29], in which the expression of the gene of interest in treated samples is represented as a fold change compared with that of control samples and normalized to an endogenous reference gene (GAPDH, GenBank AF017079) (relative quantification RQ = 1). Results are expressed as means of biological replicates ± standard deviations. Statistical analysis was performed using Student’s *t*-test (two-sided test) in Prism 8 software (GraphPad Software Inc., San Diego, CA, USA). *p* values ≤ 0.05 were considered statistically significant when ≤0.0332 (*), ≤0.0021 (**), ≤0.0002 (***), and ≤0.0001 (****).

## 3. Results

### 3.1. Transcript Assembly, Quantification and Transcriptionally Active Region (TAR) Analysis

The summary statistics of 12 cDNA libraries (six from RTX- treated and six from CTR-urinary bladder wall samples) are described in Table 1. After high-throughput sequencing, the 753,795,346 paired-end reads were processed, and 627,549,240 clean reads were obtained. Overall, more than 96% of the reads were mapped to the Sus_Scrofa.11.1.104 genome, and the mapping results displayed over 93% of uniquely aligned sequences. Using the StringTie method, the 38,378 transcriptionally active regions (TARs) were assigned and transferred to expression profiling analyses. Within the annotated TARs, 97,193 transcripts were discovered and classified. Among the annotated transcripts, 45,053 sequences were identified as protein-coding variants, 4589 as long noncoding, and 1374 other noncoding RNA annotations. The rest of the 46,177 assembled sequences had not yet been annotated in the reference genome.

### 3.2. Transcriptomic Signatures of Differentially Expressed Genes (DEGs) and Functional Annotations

**Figure 1:**
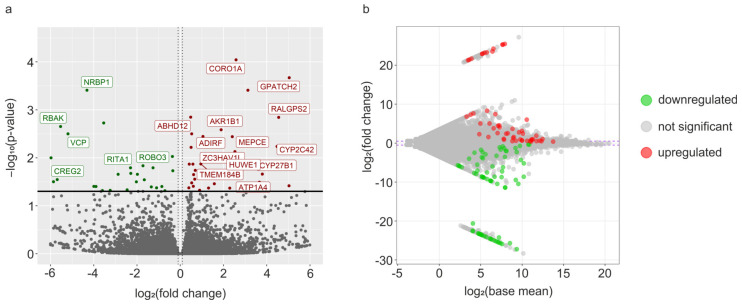
Transcriptome-wide RNA-Seq analysis and expression profiles of the differentially expressed genes (DEGs) for RTX-treated and control libraries. (**a**) The volcano plot depicts log2FC plotted against log-normalized p values. The horizontal line indicates a negative logarithmic adjusted *p* value (0.05) cut-off. Dotted double vertical lines indicate cut-off values of logFC. The square brackets comprise the symbols of the selected genes with high functional significance. (**b**) MA plot presents the logarithmic scale of the fold changes in the Y axis and the normalized expression count values in the X axis. Two horizontal dotted lines refer to the cut value of log2FC >1 and <−1. The red color illustrates upregulated DEGs; the green color represents downregulated DEGs; grey dots are nonsignificant transcripts according to DESeq2 methods.

**Figure 2:**
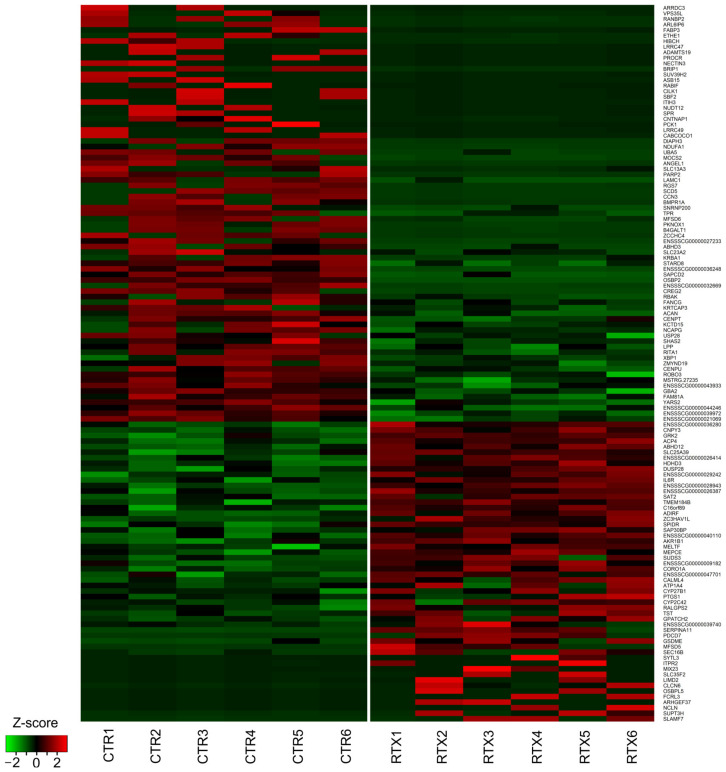
Heatmap visualization of the differentially expressed genes (DEGs) after RTX administration. Columns represent individual control (CTR) and RTX-treated (RTX) urinary bladder wall libraries; rows indicate gene symbols of DEGs. The z-score scale is applied for visualization expression values (FPKM) of each biological replicate. The red color illustrates upregulated DEGs; the green color represents downregulated DEGs.

In the present experiment, 129 differentially expressed TARs (DE-TARs) under the threshold of |log2FC| > 1 and adjusted *p* value < 0.05) were detected in RTX-urinary bladder wall libraries, as confirmed by DESseq2 (Figure 1a,b and Figure 2).

**Figure 3:**
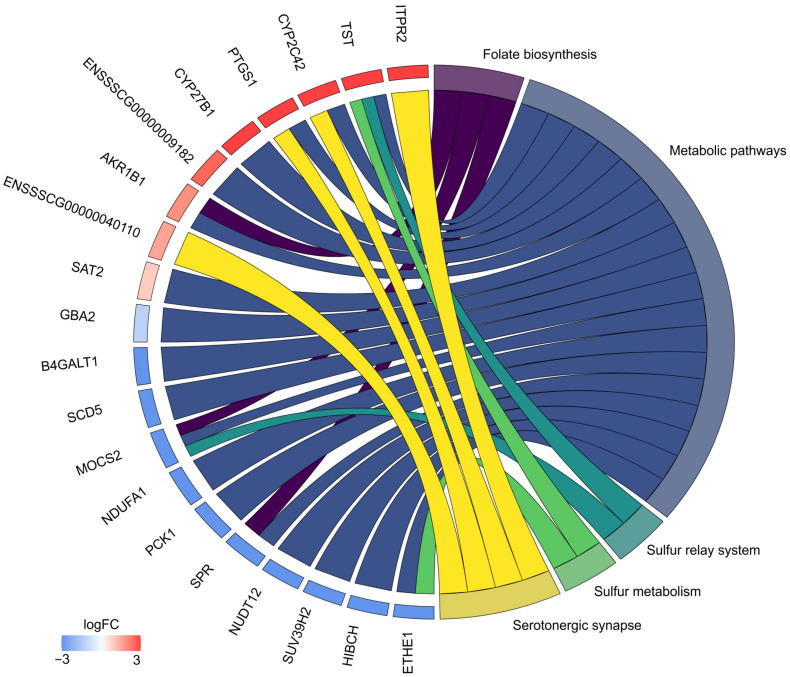
Circos plot representing five significantly enriched Kyoto Encyclopedia of Genes and Genomes (KEGG) pathways associated with differentially expressed genes (DEGs) that altered due to resiniferatoxin (RTX) intravesical administration. Gene symbols with logarithmic values (blue–red scale) of fold change (logFC) are located on the left side of the circos. Five color links merge genes with KEGG annotations (‘Folate biosynthesis’, ‘Metabolic pathways’, ‘Sulfur relay system’, ‘Sulfur metabolism’, and ‘Serotonergic synapse’) on the right side.

**Figure 4:**
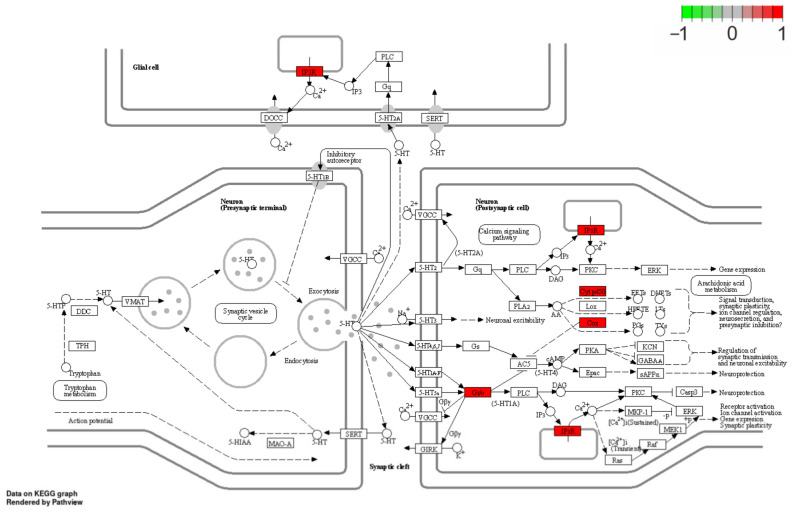
Enrichment Kyoto Encyclopedia of Genes and Genomes (KEGG) analysis of differentially expressed genes (DEGs) engaged in ‘Serotonergic synapse’. Red rectangles represent upregulated genes. Logarithmic fold change (logFC; red–green scale) describes gene expression values.

Within the TARs, 125 significant differentially expressed genes (DEGs) were identified; of them, 54 were upregulated and 71 were downregulated (Figure 2; Table 2 and Table 3). Considering the remaining four DE-TARs, three were classified as miRNA, snoRNA, lncRNA, and one was unclassified. Of 47 DEGs with an Entrez gene ID, 20 unigenes were enriched in the KEGG pathway signaling database. The KEGG assignment revealed that the DEGs were significantly categorized into five pathways (Figure 3, Table 4), including ‘Folate biosynthesis’ (Supplementary Figure S1), ‘Metabolic pathways’, ‘Sulfur relay system’, ‘Sulfur metabolism’, and ‘Serotonergic synapse’ (Figure 4). The present study identified three genes enriched in the KEGG ‘Folate biosynthesis’ pathway. One, *AKR1B1,* was overexpressed, and two genes (*SPR* and *MOCS2*) were underexpressed. Six genes assigned to the ‘Metabolic pathways’, i.e., *TST*, *CYP2C42*, *PTGS1*, *CYP27B1*, *ENSSSCG00000009182*, *AKR1B1*, and *SAT2*, were upregulated, and twelve, namely *GBA2*, *B4GALT1*, *SCD5*, *MOCS2*, *NDUFA1*, *PCK1*, *SPR*, *NUDT12*, *SUV39H2*, *HIBCH*, and *ETHE1*, were downregulated. Two altered genes, namely, upregulated *TST* and downregulated *MOCS2,* were engaged in the ‘Sulfur relay system’ KEGG pathway. Moreover, two genes were also differentially expressed in the ‘Sulfur metabolism’ pathway: again, upregulated *TST* and downregulated *ETHE1*. Furthermore, four genes were enriched in the ‘Serotoninergic synapse’ KEGG pathway: *ITPR2*, *CYP2C42*, *PTGS1*, and *ENSSSCG00000040110*. Other important DEGs were involved in apoptosis (*PDCD7*, logFC = 7.3; *GSDME*, logFC = 7.43), nerve growth and maturation (*CNTNAP1*, logFC = −23.16; *RABIF*, logFC = −23.62; *ITIH3*, logFC = −23.36), nerve regeneration (*SYTL3*, logFC = 21.28; *SLC25A39*, logFC = 0.53), and pain transmission (*GRK2*, logFC = 0.48).

### 3.3. Validation of the Results

**Figure 5:**
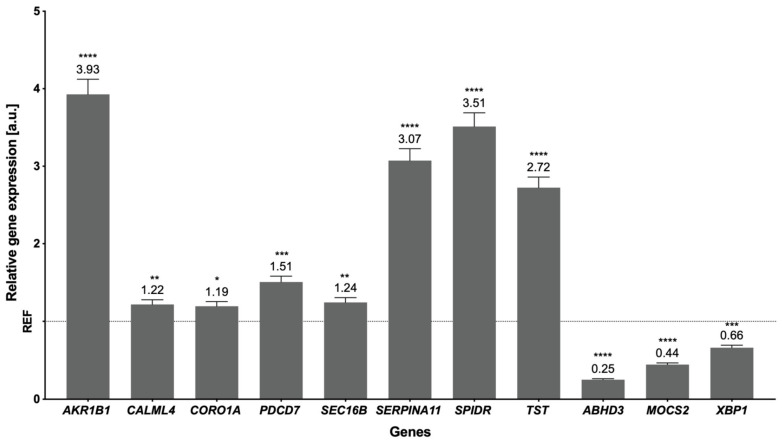
The mRNA expression of selected genes obtained through real-time PCR. The expression of endogenous control is shown as normalized to a value of 1 (REF), and the expression of validated genes indicates the changes relative to the control. The exact values of the expression are marked above the bars. *P* values ≤ 0.05 were considered statistically significant where ≤0.0332 (*), ≤0.0021 (**), ≤0.0002 (***), and ≤0.0001 (****).

The NGS method was validated using real-time PCR, which revealed the overexpression of eight genes: *AKR1B1*, *CALML4*, *CORO1A*, *PDCD7*, *SEC16B*, *SERPINA11*, *SPIDR*, and *TST*, and the underexpression of another three genes: *ABHD3*, *MOCS2* and *XBP1* (Figure 5). The genes were selected for validation following the assessment of the function, expression values, and read distribution within samples. For instance, the overexpression of *AKR1B1* and downregulation of *MOCS2* were assigned to the ‘Folate biosynthesis’ pathway. Moreover, the downregulated *MOCS2* and upregulated *TST,* revealed in RTX-treated samples, were crucial in the ‘Sulfur metabolism’ pathway. The overexpressed *PDCD7* was found to be involved in the apoptosis process and the downregulated *XBP1* in endoplasmic reticulum (ER) stress mechanisms.

## 4. Discussion

The gene expression profiling of control and RTX-treated urinary bladder samples revealed that DEGs were mostly characteristic for nervous tissue and were associated with neurodegeneration (or, on the contrary, neuroregeneration processes) and synaptic plasticity (including sensory and nociceptive transmission). The applied multistep bioinformatics identified 129 DEGs: 54 upregulated and 75 downregulated.

The primary and well-known blocking action initiated by RTX on the TRPV1 ion channel is based on its prolonged opening, which results in a massive increase in the intracellular calcium level [30]. Such a huge amount of excess calcium in the cell exerts a cytotoxic effect on the primary sensory neurons. It was found that 1 min after RTX application, mitochondria and ER undergo sudden vesiculation; approximately 5–10 min later, a nuclear membrane disruption was observed in the affected cells. This process inevitably leads to cell death and lysis, which were observed within 1–2 h after RTX treatment [31]. Nerve degeneration of small-diameter unmyelinated sensory fibers expressing TRPV1 was confirmed, for instance, in mice footpad skin after intraperitoneal injections of the toxin [32]. Concerning the urinary bladder wall, RTX intravesical therapy was followed by a marked decline in the density of TRPV1-positive nerve terminals in humans [33]. Moreover, in our previous experiment, a decrease in the number of autonomic (cholinergic and noradrenergic) nerve fibers was observed in the porcine urinary bladder after RTX intravesical instillation [18]. The results of the present study give further proof that RTX treatment induces the process of nerve degeneration. The toxin application was followed, for instance, by the upregulation of programmed cell death 7 (*PDCD7*) and gasdermin E (*GSDME*) genes, both involved in the apoptosis process [34,35]. Moreover, in the RTX-treated bladder samples, downregulation of a crucial signal transducer in ER stress, X-box binding protein 1 (*XBP1*) [36], was observed, and it was found that decreased amounts of this gene’s product (X-box binding protein 1 spliced) enhanced apoptosis [37]. Furthermore, after RTX intravesical instillation, the downregulation of several genes involved in nerve growth and maturation was observed, including contactin associated protein 1 (*CNTNAP1*), RAB interacting factor (*RABIF*), interalpha–trypsin inhibitor heavy chain 3 (*ITIH3*), 3-hydroxyisobutyryl-CoA hydrolase (*HIBCH*), ubiquitin-like modifier activating enzyme 5 (*UBA5*), cellular communication network factor 3 (*CCN3*), solute carrier family 23 member 2 (*SLC23A2*), SET binding factor 2 (*SBF2*), and abhydrolase domain containing 3 phospholipase (*ABHD3*). The first of the abovementioned underexpressed genes, *CNTNAP1*, encodes contactin-associated protein, which is essential for the development of paranodal junctions as well as proper radial and longitudinal organization of myelinated nerve fibers [38,39]. *CNTNAP1*-associated disturbances are one of the most serious hypomyelinating disorders of both the central and peripheral nervous systems [40,41]. *RABIF* encodes a member of small GTP-binding proteins that participate in the control of intracellular vesicular transport. It was found that Rab GTPases are involved in the process of neurite outgrowth and axon specification [42]. Another downregulated gene, *ITIH3*, encodes the heavy chain of the prealpha–trypsin inhibitor complex, which, through its ability to bind hyaluronic acid, normally stabilizes the extracellular matrix. It was discovered that polymorphism of *ITIH3* may alter protein expression and neurodevelopment [43]. The next gene, *HIBCH*, is thought to be one of the regulators of neuronal stability [44], and its mutations may lead, for instance, to progressive infantile neurodegeneration [45]. Another gene downregulated in the RTX-treated bladder wall, *UBA5*, encodes a member of the E1-like ubiquitin-activating enzyme family, which promotes the post-translational modification of the proteins. UBA5 is also known to play a crucial role in neurodevelopment [46]. Mutations of this gene may result in hypomyelination and neurodegeneration [47]. *CCN3* encodes an immediate–early protein involved in numerous cellular processes such as proliferation, adhesion, migration, differentiation, and survival [48]. It has been also found that CCN3 is engaged in neuroimmune responses, myelin regeneration in nerve cells [49,50] and neurite growth [51]. Moreover, RTX treatment contributed to the downregulation of the *SLC23A2* gene, encoding the sodium-dependent vitamin C transporter 2 (SVCT2). This protein is engaged in the regeneration process after peripheral nerve injury. It was found that SVCT2 deficiency reduces the amount of ascorbic acid in the peripheral nerves, leading to hypomyelination and collagen-containing extracellular matrix deficits [52]. A previous study revealed that the *SBF2* gene is involved in controlling phosphoinositide signaling, which participates in the myelination process, and its mutations were found in autosomal recessive hereditary motor and sensory neuropathy (also known as Charcot–Marie–Tooth disease) [53]. Furthermore, Cunningham, et al. [54] revealed the impact of *SBF2* on the development of taxane-induced peripheral neuropathy. Finally, RTX treatment evoked the downregulation of *ABHD3*, a gene encoding phospholipase that may play a role in the remodeling of phospholipids [55], which are essential constituent molecules of both neuronal cell bodies and neurites [56]. It is well known that the intricate cooperation between lipids (including phospholipids) and proteins greatly alters presynaptic activity [57].

Interestingly, apart from the DEGs reflecting the ongoing nerve degeneration caused by RTX application, the present study also revealed these indications of the initiation of neuroprotective mechanisms and nerve regeneration process. For instance, the toxin treatment resulted in the upregulation of synaptotagmin-like 3 (*SYTL3*), solute carrier family 25 member 39 (*SLC25A39*), cytochrome p450 family 27 subfamily B member 1 (*CYP27B1*), or Chloride voltage-gated channel 6 (*CLCN6*). It was found that *SYTL3*, by regulating the expression of matrix metalloproteinase, plays a crucial role in neuronal migration and development [58]. Moreover, the *SYTL3* translation product, synaptotagmin-like protein, regulates vesicle trafficking and docking and thus synaptic neurotransmitter release [59]. *SLC25A39* encodes the mitochondrial transporter of the SLC25 family [60]. It was found that the Drosophila homolog of *SLC25A39* promotes neuronal survival [61]; thus, the upregulation of *SLC25A39* may also be considered a neuroprotective mechanism in mammals. Another gene upregulated in the RTX-treated samples, *CYP27B1*, is also associated with mitochondria function, and it encodes the 25-hydroxyvitamin D-1α-hydroxylase enzyme, which involved in vitamin D metabolism and is needed for converting inactive 25-OHD to biologically active 1,25-(OH)2D [62]. Vitamin D is a well-known antioxidant that exerts its neuroprotective effect, among others, by decreasing oxidative stress, regulating calcium metabolism, or participating in immunomodulation or glutamatergic transmission [63]. Furthermore, RTX intravesical administration induced the upregulation of chloride voltage-gated channel 6 (*CLCN6*), which encodes a member of the voltage-dependent chloride channel protein 6 (CLC6), functioning as a Cl^−^/H^+^-exchanger in the endolysosomal pathway of mammalian neurons [64,65]. The exact role of CLC6 is not known, but this protein is most probably engaged in ion homeostasis and pH regulation of late endosomes. Polovitskaya et al. [66] revealed that mutations in CLCN6 are closely related to some neurological disorders.

As previously mentioned, the main rationale for using RTX to treat LUTSs, such as overactive bladder (OAB), is to diminish the nociceptive bladder input carried to the spinal cord by unmyelinated C fibers [67]. According to the obtained results, RTX treatment led to the identification of DEGs strongly involved in the pain transmission process. It is worth emphasizing that toxin administration was followed by the upregulation of G protein-coupled receptor kinase 2 (*GRK2*). The GRK2 protein encoded by this gene is well known to promote the desensitization of G protein-coupled receptors engaged in nociceptive transmission [68]. It was found that the downregulation of *GRK2* leads to increased pain receptors’ signaling and prolongs the prostaglandin E2-induced hyperalgesia [69]. Moreover, in mice, a low level of GRK2 resulted in the enhancement in both the mechanical and thermal hyperalgesia induced by epinephrine [70]. On the other hand, Wang et al. [71] revealed that the increase in GRK2 inhibited chronic hyperalgesia. Furthermore, it was proven that overexpression of GRK2 strikingly weakens the sensitization of TRPV1 receptors [72]. Thus, the present findings suggest that RTX exerts its blocking action on TRPV1 by upregulation of *GRK2*.

Remarkably, RTX instillation was also followed by the downregulation of sepiapterin reductase (*SPR*). Sepiapterin reductase, encoded by SPR, catalyzes the reduction of sepiapterin to tetrahydrobiopterin (BH4), a molecule that, when upregulated, results in a higher sensitivity to pain stimuli [73]. It should be emphasized that the inhibition of SPR in the BH4 pathway was proposed as one of the possible treatment options to alleviate chronic pain in humans. The BH4 pathway is usually activated by proinflammatory factors and leads to the generation of several bioactive molecules with the ability to modify neuropathic pain sensitivity [74]. Therefore, the downregulation of *SPR* observed in RTX-treated bladder should be considered an advantageous result regarding the treatment of LUTSs characterized by an increase in bladder pain sensation. Furthermore, BH4 acts as a cofactor for the aromatic amino acid hydroxylases involved in the synthesis of several neurotransmitters, such as serotonin, noradrenaline, dopamine, and nitric oxide [75,76]. Thus, the underexpression of *SPR* may additionally represent the plastic changes induced in the peripheral nerves after RTX intravesical instillation. Importantly, *SPR* also participates in folate (vitamin B9) formation and metabolism. The present study identified three genes enriched in the KEGG ‘Folate biosynthesis’ pathway. Two of these genes, SPR and molybdenum cofactor synthesis 2 (*MOCS2*), were underexpressed, and one, aldo-keto reductase family 1 member B (*AKR1B1*), was overexpressed. Folate is possibly the most well-recognized participant in proper neural tube formation, and its supplementation before and during early pregnancy helps to prevent neural tube defects [77]. Vitamin B9 deficiency has been correlated with several neurodegenerative disorders, including Parkinson’s and Alzheimer’s diseases [78]. The role of folate in the regeneration and repair of injured neural tissues has been widely investigated. Iskandar et al. [79] found that folate stimulates sensory spinal axons regrowth in vivo, and its neurodegenerative properties are not limited to the early embryonic period but are still effective in the adult central nervous system. Moreover, it was proven that folate treatment, by decreasing matrix metalloproteinase-2, reduces neuropathic pain and enhances functional recovery after spinal cord contusion [80]. It is reasonable to assume that the modified gene expression within the KEGG’s ‘Folate biosynthesis’ pathway, in conjunction with the upregulation of *GRK2*, could enhance the antinociceptive effect exerted by RTX on TRPV1 in LUTSs associated with exaggerated pain transmission, such as interstitial cystitis [81].

Intriguingly, several DEGs revealed in the RTX-treated urinary bladder wall encoded molecules involved in synaptic signaling. Namely, four genes were enriched in the ‘Serotoninergic synapse’ KEGG pathway: cytochrome P450 2C42 (*CYP2C42*), ENSSSCG00000040110, prostaglandin-endoperoxide synthase 1 (*PTGS1*, also known as *COX1*), and inositol 1,4,5-trisphosphate receptor type 2 (*ITPR2*). Serotonin (5-HT) is a neurotransmitter found in the central and peripheral nervous systems and is well known to participate in pain transmission [82]. Moreover, 5-HT receptors play a crucial role in the control of the micturition reflex. The action of 5-HT on both pain sensation and micturition reflex differs depending on the subtype of the activated receptor. So far, 14 types of structurally different 5-HT receptors, grouped into seven families (5-HT1–7), have been recognized. The genes upregulated in the RTX-treated bladder wall were found to be involved in pathways triggered by the activation of 5-HT1 (a–f), 5-HT2, and 5HT5a receptors. The 5-HT1 and 5-HT2 receptors participate in micturition control [83]; however, their activation seems to exert the opposing effect. In rats, the excitation of 5-HT1A receptors stimulates micturition at the supraspinal level [84]; on the contrary, the excitation of 5-HT2 receptors inhibits the micturition reflex [85]. Regarding 5HT5a receptors, their activation, at least at the spinal level, exerts an antinociceptive impact in several pain models in rodents [86]. Therefore, the results of the present study indicate that RTX may exert its therapeutic effect by influencing 5-HT transmission.

Among the findings of this study, two altered genes annotated to the ‘Sulfur relay system’ KEGG pathway were identified, namely, the previously described downregulated *MOCS2* and upregulated thiosulfate sulfurtransferase (*TST*). Moreover, two genes were also differentially expressed in the ‘Sulfur metabolism’ pathway again: upregulated *TST* and downregulated ETHE1 persulfide dioxygenase (*ETHE1*). Both *TST* and *ETHE1* encode mitochondrial enzymes that catalyze the transfer of sulfur in several molecular pathways [87,88]. *ETHE1* plays an essential role in hydrogen sulfide catabolism in the mitochondrial matrix by metabolizing hydrogen sulfide and preventing the accumulation of supraphysiological H2S levels, which has toxic effects [88]. Currently, H2S is also considered a physiologically active messenger and in the urinary bladder; this gaseous signaling molecule is a potential chemical entity for the activation of transient receptor potential ankyrin 1 (TRPA1) channels [89]. TRPA1, the same as TRPV1, belongs to the transient receptor potential (TRP) superfamily and is mainly expressed in the primary sensory neurons but is also found in the urothelium, some interstitial cells, and detrusor muscle [90]. A study in rats revealed that H2S induces detrusor overactivity, increased micturition frequency, and most likely bladder inflammation [91]. Therefore, again, increased catabolism of H2S, caused by the downregulation of *ETHE1*, should be considered an advantageous effect from the perspective of LUTS treatment.

It must be mentioned that RTX treatment was also followed by the upregulation of abhydrolase domain containing 12, lysophospholipase (*ABHD12*), a gene encoding monoacylglycerol lipase catalyzing the hydrolysis of 2-arachidonoyl glycerol (2-AG), which is the primary endocannabinoid lipid transmitter acting on cannabinoid receptors CB1 and CB2. The binding of 2-AG to these receptors exerts an analgesic effect; thus, endocannabinoids were extensively investigated in studies concerning bladder pain control [92]. The observed upregulation of *ABHD12*, and thus increased hydrolysis of 2-AG, suggests that RTX’s analgesic effect is not mediated through CB1 and CB2 activation. On the other hand, it was found that genetically modified mice devoid of ABHD12 exhibit abnormal neurobehavior due to a dysregulated lysophospholipase pathway [93], suggesting that ABHD12 may have a neuroprotective role.

The upregulation of spermidine/spermine N(1)-acetyltransferase 2 (*SAT2*) and the downregulation of regulator of G protein signaling 7 (*RGS7*) in the bladder wall suggest that the toxin also affects glutamate and GABAergic transmission. SAT2 is a transporter responsible for presynaptic glutamine uptake and, therefore, glutamate resynthesis [94]. It was found that glutaminergic transmission plays a significant facilitatory role in the afferent transduction of the micturition reflex and is involved in bladder nociception [95]. RGS7 modulates synaptic plasticity and GABA signaling in the central nervous system [96,97]. GABA exerts peripherally mediated inhibition of urinary bladder detrusor muscle contractility in human [98]. Thus, alterations in *SAT2* and *RGS7* expression may modulate the contractility and pain sensation in the bladder. Moreover, in the cultured cell lines, RGS7 reduced muscarinic M3 [99,100] and 5-HT2C receptor activation [101]. In this regard, underexpression of *RGS7* may lead to the activation of M3 receptors, which enhance contractions of the urinary bladder wall [102], but it may stimulate 5-HT2 receptors, which are responsible for inhibiting the micturition reflex [85].

## 5. Conclusions

To the best of our knowledge, this is the first study concerning the targeted gene expression profiling and pathway analysis of control and intravesically RTX-treated porcine urinary bladder walls. The revealed DEGs were assigned to neurodegeneration or neuroprotection processes and nociceptive transmission. Interestingly, several transcripts were found to be engaged in the neuromodulation of synaptic plasticity by affecting 5-HT, H2S, glutamate, or GABA transmission. Evidence from this study implies that RTX exerts its therapeutic, antinociceptive effect not only by acting on TRPV1 receptors. It should be noted that the analyzed urinary bladder wall samples contained all the layers of the bladder wall; thus, the present study did not allow for differentiation of the changes within the subcompartments such as the urothelium, lamina propria, detrusor, adventitia, or serosa. Moreover, it cannot be excluded that the number of cells within each of these subcompartments might have influenced the gene expression profile. Nevertheless, the transcriptomic identification of DEGs sheds light on how RTX affects bladder function and broadens the potential target points for clinical interventions. Therefore, we hope that this research lays the groundwork for studies and clinical trials regarding the possible use of RTX not only in the treatment of urological dysfunctions but also in other diseases associated with exaggerated pain sensation.

## Figures and Tables

**Table 1:** Summary of statistical metrics for the RNA-Seq libraries; CTR refers to control samples; RTX refers to the biological replicates of the experimental (RTX-treated) urinary bladder wall samples; “Raw reads“ refers to the number of reads obtained from sequencing protocol; “Trimmed reads” refers to reads that passed the quality control procedure; “Mapped reads” refers to all reads aligned to the reference genome; “Uniquely mapped reads” refers to reads that were mapped to a unique (only one) location of the reference genome; “Multimapped reads” refers to reads aligned to more than one locus on the reference genome; “Reads mapped with too many loci” refers to the reads mapped more than 20 multiple loci on the reference genome.

RNA-Seq Libraries	Raw Reads	Trimmed Reads	Mapped Reads	Uniquely Mapped Reads	Multi-Mapped Reads	Too Many Loci
CTR1	63,450,432	53,906,522	51,882,476	50,773,306	1,065,278	43,892
CTR2	63,018,144	52,441,270	50,719,276	49,751,418	941,066	26,792
CTR3	63,747,778	54,451,852	51,875,622	50,745,164	1,099,474	30,984
CTR4	63,650,872	53,024,696	50,606,392	49,420,902	1,156,814	28,676
CTR5	64,447,908	53,525,868	52,020,672	51,016,528	974,894	29,250
CTR6	61,433,064	51,078,746	49,682,244	48,575,512	1,074,524	32,208
RTX1	62,806,456	51,157,178	49,658,774	48,627,582	996,796	34,396
RTX2	61,901,726	51,796,642	48,881,448	47,799,714	1,050,198	31,536
RTX3	62,638,642	52,315,264	49,903,750	48,713,644	1,162,622	27,484
RTX4	62,536,662	51,887,614	50,170,978	48,818,308	1,313,042	39,628
RTX5	62,688,462	51,465,770	50,012,444	48,808,958	1,171,510	31,976
RTX6	61,475,200	50,497,818	47,752,176	46,484,026	1,229,930	38,220

**Table 2:** Significant upregulated differentially expressed genes (DEGs) between RTX-treated (RTX) and control (CTR) libraries.

Gene ID	Gene Name	log2FoldChange	*p*-Adjusted
MSTRG.18558	*SLAMF7*	25.47	7.96 × 10^−14^
MSTRG.24634	*SUPT3H*	25.30	9.68 × 10^−14^
MSTRG.14735	*NCLN*	25.29	9.68 × 10^−14^
MSTRG.15606	*ARHGEF37*	24.25	1.13 × 10^−12^
MSTRG.18613	*FCRL3*	23.54	5.68 × 10^−12^
MSTRG.13448	*OSBPL5*	23.30	9.30 × 10^−12^
MSTRG.22604	*CLCN6*	23.18	9.71 × 10^−18^
MSTRG.4591	*LIMD2*	22.96	2.08 × 10^−11^
MSTRG.27341	*SLC35F2*	22.92	2.22 × 10^−11^
MSTRG.7236	*MIX23*	21.70	4.42 × 10^−10^
MSTRG.20205	*ITPR2*	21.42	8.65 × 10^−10^
MSTRG.137	*SYTL3*	21.28	1.17 × 10^−9^
MSTRG.28195	*SEC16B*	8.31	0.0036
MSTRG.19812	*MFSD5*	7.62	0.0403
MSTRG.13163	*GSDME*	7.43	0.0105
MSTRG.1415	*PDCD7*	7.30	0.0436
ENSSSCG00000002479	*SERPINA11*	6.81	0.0006
MSTRG.13644	*ENSSSCG00000039740*	6.03	0.0003
MSTRG.2732	*GPATCH2*	5.04	0.0002
MSTRG.19650	*TST*	5.03	0.0389
MSTRG.28205	*RALGPS2*	4.55	0.0015
MSTRG.9190	*CYP2C42*	4.48	0.0059
MSTRG.2418	*PTGS1*	4.06	0.0356
MSTRG.19993	*CYP27B1*	3.79	0.0221
MSTRG.18574	*ATP1A4*	3.66	0.0328
MSTRG.1486	*CALML4*	3.13	0.0004
MSTRG.29522	*ENSSSCG00000047701*	2.78	0.0214
MSTRG.16166	*CORO1A*	2.58	9.19 × 10^−5^
MSTRG.26707	*ENSSSCG00000009182*	2.53	0.0075
MSTRG.8288	*SUDS3*	2.46	0.0018
MSTRG.15899	*MEPCE*	2.41	0.0037
MSTRG.7148	*MELTF*	2.29	0.0434
MSTRG.12780	*AKR1B1*	1.89	0.0026
MSTRG.4949	*ENSSSCG00000040110*	1.58	0.0015
MSTRG.4430	*SAP30BP*	1.58	0.0352
MSTRG.18428	*SPIDR*	1.31	0.0434
MSTRG.12716	*ZC3HAV1L*	1.09	0.0101
MSTRG.9033	*ADIRF*	1.05	0.0036
MSTRG.16386	*C16orf89*	1.02	0.0221
MSTRG.19607	*TMEM184B*	0.95	0.0136
MSTRG.5521	*SAT2*	0.89	0.0483
MSTRG.14799	*ENSSSCG00000026387*	0.89	0.0224
MSTRG.21677	*ENSSSCG00000028943*	0.85	2.31 × 10^−15^
MSTRG.18711	*IL6R*	0.69	0.0185
MSTRG.22744	*ENSSSCG00000029242*	0.67	0.0283
MSTRG.10953	*DUSP28*	0.62	0.0227
MSTRG.2338	*HDHD3*	0.60	0.0399
MSTRG.11546	*ENSSSCG00000026414*	0.59	0.0137
MSTRG.4676	*SLC25A39*	0.53	0.0337
MSTRG.11916	*ABHD12*	0.53	0.0032
MSTRG.22082	*ACP4*	0.50	0.0062
MSTRG.13531	*GRK2*	0.48	0.0014
MSTRG.24570	*CNPY3*	0.42	0.0137
MSTRG.8629	*ENSSSCG00000036280*	0.40	0.0428

**Table 3:** Significantly downregulated differentially expressed genes (DEGs) between RTX-treated (RTX) and control (CTR) libraries.

Gene ID	Gene Name	log2FoldChange	*p*−Adjusted
MSTRG.15089	*ARRDC3*	−27.24	1.70 × 10^−15^
MSTRG.16274	*VPS35L*	−25.90	5.74 × 10^−14^
MSTRG.16760	*RANBP2*	−25.69	5.74 × 10^−14^
MSTRG.10097	*ARL6IP6*	−25.69	5.74 × 10^−14^
MSTRG.22915	*FABP3*	−25.51	7.96 × 10^−14^
MSTRG.21796	*ETHE1*	−25.41	8.44 × 10^−14^
MSTRG.10408	*HIBCH*	−25.18	1.23 × 10^−13^
MSTRG.22503	*LRRC47*	−24.99	1.93 × 10^−13^
MSTRG.15319	*ADAMTS19*	−24.68	4.27 × 10^−13^
MSTRG.12082	*PROCR*	−24.61	4.90 × 10^−13^
MSTRG.7356	*NECTIN3*	−24.51	5.96 × 10^−13^
MSTRG.5103	*BRIP1*	−24.23	1.13 × 10^−12^
MSTRG.3245	*SUV39H2*	−24.09	1.57 × 10^−12^
MSTRG.12889	*ASB15*	−23.88	2.67 × 10^−12^
MSTRG.2979	*RABIF*	−23.62	5.12 × 10^−12^
MSTRG.24689	*CILK1*	−23.55	5.68 × 10^−12^
MSTRG.14163	*SBF2*	−23.49	6.31 × 10^−12^
MSTRG.6267	*ITIH3*	−23.36	8.48 × 10^−12^
MSTRG.15168	*NUDT12*	−23.34	8.76 × 10^−12^
MSTRG.17031	*SPR*	−23.26	9.96 × 10^−12^
MSTRG.4745	*CNTNAP1*	−23.16	5.74 × 10^−14^
MSTRG.12395	*PCK1*	−22.59	5.18 × 10^−11^
MSTRG.1522	*LRRC49*	−22.53	5.88 × 10^−11^
MSTRG.8812	*CABCOCO1*	−20.62	5.39 × 10^−9^
MSTRG.3953	*DIAPH3*	−11.41	2.52 × 10^−5^
MSTRG.29959	*NDUFA1*	−11.39	9.13 × 10^−7^
MSTRG.6637	*UBA5*	−10.47	7.95 × 10^−6^
MSTRG.11212	*MOCS2*	−10.45	7.86 × 10^−8^
MSTRG.25491	*ANGEL1*	−9.75	0.0013
MSTRG.12280	*SLC13A3*	−9.71	0.0151
MSTRG.25217	*PARP2*	−9.64	0.0018
MSTRG.28269	*LAMC1*	−9.45	2.65 × 10^−7^
MSTRG.2788	*RGS7*	−9.23	0.0018
MSTRG.26818	*SCD5*	−8.80	0.0032
MSTRG.17952	*CCN3*	−8.80	0.0033
MSTRG.9028	*BMPR1A*	−8.78	0.0092
MSTRG.16749	*SNRNP200*	−8.72	7.95 × 10^−6^
MSTRG.28308	*TPR*	−8.64	9.15 × 10^−7^
MSTRG.10410	*MFSD6*	−8.63	1.35 × 10^−5^
MSTRG.7732	*PKNOX1*	−8.61	0.0003
MSTRG.3103	*B4GALT1*	−8.59	0.0037
MSTRG.25925	*ZCCHC4*	−7.93	0.0150
MSTRG.5125	*ENSSSCG00000027233*	−7.67	0.0005
MSTRG.23226	*ABHD3*	−7.52	0.0021
MSTRG.11786	*SLC23A2*	−7.27	0.0007
MSTRG.13311	*KRBA1*	−7.08	0.0293
MSTRG.29606	*STARD8*	−6.20	7.95 × 10^−6^
MSTRG.4433	*ENSSSCG00000036248*	−6.17	0.0004
MSTRG.28999	*SAPCD2*	−5.98	0.0101
MSTRG.8518	*OSBP2*	−5.88	1.08 × 10^−5^
ENSSSCG00000032669	*ENSSSCG00000032669*	−5.86	0.0319
ENSSSCG00000008166	*CREG2*	−5.69	0.0289
MSTRG.15782	*RBAK*	−5.53	0.0023
MSTRG.2091	*FANCG*	−5.19	0.0032
MSTRG.17449	*KRTCAP3*	−4.32	0.0004
MSTRG.24821	*ACAN*	−4.00	0.0400
MSTRG.21369	*CENPT*	−3.90	0.0403
MSTRG.21518	*KCTD15*	−3.60	0.0488
MSTRG.25897	*NCAPG*	−3.54	0.0019
MSTRG.27421	*USP28*	−3.24	0.0485
MSTRG.17940	*SHAS2*	−2.88	0.0224
MSTRG.7109	*LPP*	−2.46	0.0473
MSTRG.8347	*RITA1*	−2.30	0.0164
MSTRG.8476	*XBP1*	−2.29	0.0214
MSTRG.28939	*ZMYND19*	−2.03	0.0321
MSTRG.9945	*CENPU*	−1.98	0.0224
MSTRG.27587	*ROBO3*	−1.73	0.0149
MSTRG.27235	*NA*	−1.66	0.0291
MSTRG.11646	*ENSSSCG00000043933*	−1.34	0.0410
MSTRG.2104	*GBA2*	−1.26	0.0164
MSTRG.924	*FAM81A*	−1.09	0.0429
MSTRG.20134	*YARS2*	−0.85	0.0403
MSTRG.3339	*ENSSSCG00000044246*	−0.71	0.0494
MSTRG.2389	*ENSSSCG00000039972*	−0.36	0.0095
MSTRG.24246	*ENSSSCG00000021069*	−0.34	0.0189

**Table 4:** The results of the Kyoto Encyclopedia of Genes and Genomes (KEGG) pathways for differentially expressed genes (DEGs) detected in resiniferatoxin (RTX)-treated samples.

Term ID	KEGG Name	*p*-Adjusted *	Gene Number	Gene Symbol
KEGG:00790	Folate biosynthesis	0.0224	3	SPR, MOCS2, AKR1B1
KEGG:01100	Metabolic pathways	0.0621	18	ETHE1, HIBCH, SUV39H2, NUDT12, SPR, PCK1, NDUFA1, MOCS2, SCD5, B4GALT1, GBA2, SAT2, AKR1B1, ENSSSCG00000009182, CYP27B1, PTGS1, CYP2C42, TST
KEGG:04122	Sulfur relay system	0.0845	2	MOCS2,TST
KEGG:00920	Sulfur metabolism	0.1052	2	ETHE1,TST
KEGG:04726	Serotonergic synapse	0.2291	4	ENSSSCG00000040110, PTGS1, CYP2C42, ITPR2

* calculated with the g:SCS algorithm.

## Data Availability

The datasets analyzed during the current study are available from the ENA database under accession number: PRJEB55778 (https://www.ebi.ac.uk/ena, accessed on 30 September 2022).

## Supplementary Materials

The following supporting information can be downloaded at: https://www.mdpi.com/article/10.3390/cells12030462/s1, Figure S1: Enrichment Kyoto Encyclopedia of Genes and Genomes (KEGG) analysis of differentially expressed genes (DEGs) engaged in the ‘Folate biosynthesis’ signaling pathway; Table S1: The list of primers used for real-time PCR.
