# Supplementary material for: Molecular Influence of Resiniferatoxin on the Urinary Bladder Wall Based on Differential Gene Expression Profiling
Source: Cells. 2023 Jan 31;12(3):462. doi: 10.3390/cells12030462 (PMC9914288; doi:10.3390/cells12030462)
Supplement: Supplementary file 1 [file cells-12-00462-s001.zip › cells-2170188-supplementary.pdf]

Table S1. The list of primers used for Real-Time PCR

Name	Forward sequence: (5' to 3')	Reverse sequence: (5' to 3')
AKR1B1	CTGTGAAGGTGGCCATTGAC	GTGGGCCAGTGGATAAGGTA
CALML4	CTGACTGTGATGAGGTGCCT	CATGAGTTTGGACCGCAGTT
CORO1A	GACAACGTGATCCTGGTGTG	CTTCTCCGCTACAATGGTGC
PDCD7	CGAGAGCAGGAAATTGACCG	TTCTCCAAGGCCCGAAGAAT
SEC16B	CGAAGCTGCTCTACTACGGA	TCCCTGACATGAGCTGGAAG
SERPINA11	CCTACCAGACCCAGAAGCAA	CTACCTGTGCCATTTTCCCG
SPIDR	TCACCCGAAAGCCAGGTTAT	AGTACACTCTCTGCACCACC
TST	GCTCAGTCAACATGCCCTTC	ACCAGGAGCCGTCATAGATG
ABHD3	CGGTTCGGGAGTAGGCTTAT	AGGGGCTTGGAAGTGATGAA
MOCS2	GCTCCTCCTGTTGCAATCAG	ACCGCAGACAGGAGAAATCA
XBP1	GCCTCCCCTTCTTCATCACT	TTTCTCTGAGGGGCTGGAAG

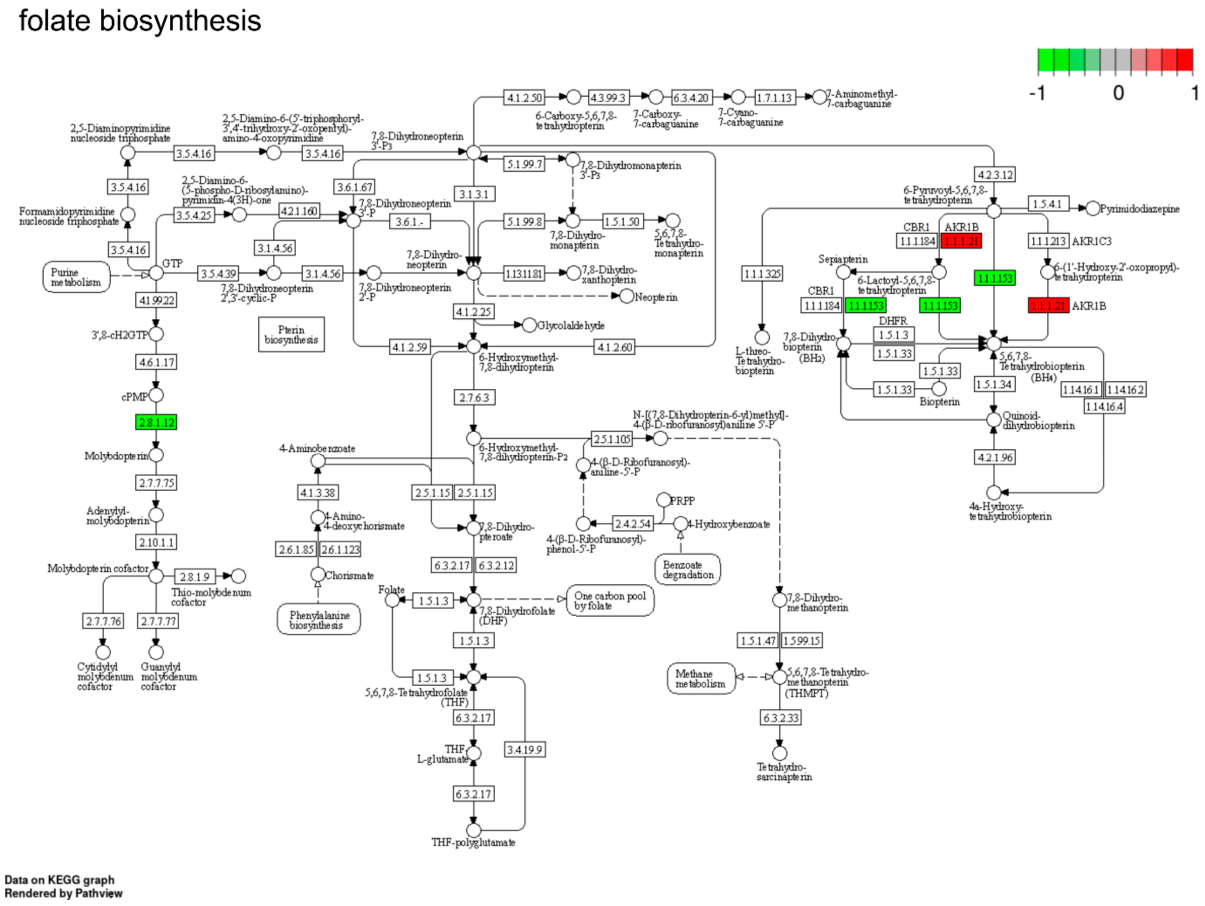

Figure S1. Enrichment Kyoto Encyclopedia of Genes and Genomes (KEGG) analysis of differentially expressed genes (DEGs) engaged in the ‘Folate biosynthesis’ signaling pathway. Red and green rectangles present upregulated and downregulated genes, respectively. Logarithmic fold change (logFC; red-green scale) values describe gene expression values.
